# Sources and reservoirs of *Staphylococcus capitis* NRCS-A inside a NICU

**DOI:** 10.1186/s13756-019-0616-1

**Published:** 2019-10-17

**Authors:** Marine Butin, Yann Dumont, Alice Monteix, Aurane Raphard, Christine Roques, Patricia Martins Simoes, Jean-Charles Picaud, Frédéric Laurent

**Affiliations:** 1 Centre International de Recherche en Infectiologie (CIRI), INSERM U1111, CNRS UMR5308, Ecole Normale Supérieure de Lyon, Université Claude Bernard Lyon 1, 46 Allée d’Italie, 69364 Lyon Cedex 07, France; 2 Réanimation Néonatale, Hôpital Femme Mère Enfant, Hospices Civils de Lyon, 59 boulevard Pinel, 69500 Bron, France; 3 Institut des Agents Infectieux, Centre National de Référence des Staphylocoques, Hôpital de la Croix Rousse, Hospices Civils de Lyon, 104 grande rue de la Croix Rousse, 69004 Lyon, France; 4 Laboratoire de Génie Chimique UMR 5503, Université de Toulouse, CNRS, INPT, UPS, Faculté des Sciences Pharmaceutiques, 35 chemin des maraîchers, 31062 Toulouse cedex 4, France; 5 Réanimation Néonatale, Hôpital de la Croix Rousse, Hospices Civils de Lyon, 104 grande rue de la Croix Rousse, 69004 Lyon, France; 6 CarMeN, INSERM U1060, INRA U1397, Université de Lyon, 165 Chemin du Grand Revoyet, 69310 Pierre Bénite, France; 7 Département de Microbiologie et Mycologie, Institut des Sciences Pharmaceutiques et Biologiques de Lyon, Université de Lyon, 6 Avenue Rockefeller, 69008 Lyon, France

**Keywords:** *Staphylococcus capitis*, NRCS-A, Neonatal ICUs, Sepsis, Incubators, Environment

## Abstract

**Background:**

The methicillin-resistant clone *Staphylococcus capitis* NRCS-A, involved in sepsis in neonatal intensive care units (NICUs) worldwide, is able to persist and spread in NICUs, suggesting the presence of reservoirs inside each setting. The purpose of the present study was to identify these reservoirs and to investigate the cycle of transmission of NRCS-A in one NICU.

**Methods:**

In a single institution study, NRCS-A was sought in 106 consecutive vaginal samples of pregnant women to identify a potential source of NRCS-A importation into the NICU. Additionally NICU caregivers and environmental including incubators were tested to identify putative secondary reservoirs. Finally, the efficacy of disinfection procedure in the elimination of NRCS-A from incubators was evaluated.

**Results:**

No *S. capitis* was isolated from vaginal samples of pregnant women. Three of the 21 tested caregivers (14%) carried *S. capitis* on their hands, but none remain positive after a five-day wash-out period outside NICU. Moreover, the clone NRCS-A persisted during six consecutive weeks in the NICU environment, but none of the sampled sites was constantly contaminated. Finally in our before/after disinfection study, all of 16 incubators were colonized before disinfection and 10 (62%) incubators remained colonized with NRCS-A after the disinfection procedure.

**Conclusions:**

The partial ineffectiveness of incubators’ disinfection procedures is responsible for persistence of NRCS-A inside a NICU, and the passive hand contamination of caregivers could be involved in the inter-patient transmission of *S. capitis*.

## Background

Nosocomial late onset sepsis, mostly due to coagulase negative staphylococci (CoNS), is a major cause of mortality and morbidity in hospitalized neonates, especially in very low birth weight infants. The involvement of *Staphylococcus capitis* species in these infections has been reported by numerous authors, either sporadically or in local epidemics [1–3].

Recently, a specific clone belonging to this species, named NRCS-A, has emerged as a major pathogen in hospitalized neonates [4, 5]. Studies have highlighted the worldwide distribution of this clone and its specific antimicrobial multidrug resistant profile, including resistance to the usual first-line antibiotics used in neonatal intensive care units (NICUs), i. e. vancomycin and aminoglycosides. Furthermore, higher morbidity of *S. capitis* NRCS-A-related sepsis when compared to other CoNS has been reported [6].

Despite the worldwide distribution and endemicity of the clone NRCS-A, poorly is known about the source of contamination of neonates. Its specific affinity for the neonates may suggest a maternal carriage responsible for a maternal-fetal contamination. In addition, epidemiological investigations have shown that once present in a NICU, the clone has a large propensity to persist and to reach high prevalence within the setting [5, 7]. This suggests the presence of reservoirs inside each NICU, either in the hospital environment, or in asymptomatic carriers.

A better understanding of the origin, ecological niches and reservoirs of *S. capitis* NRCS-A inside a NICU should help in managing and controlling its local and global spread. The objectives of the present study were: i) to screen pregnant women as a potential source of *S. capitis* contamination, ii) to screen *S. capitis* carriage among NICU caregivers, iii) to identify the distribution and environmental persistence of this clone inside a NICU. On the basis of our results, we also aimed to evaluate the efficacy of incubators disinfection procedure.

## Methods

### Study setting and method of sampling and identification of S. capitis

The study was conducted in the Neonatal Intensive Care Unit and in the Institute of Infectious Agents, at Hospital Croix Rousse, Hospices Civils de Lyon, France. Environmental surfaces were sampled using flocked nylon swabs (ESwab, Copan®) which have been showed to enhance efficiency and recovery of inoculum [8]. Human clinical samples were performed using dry swabs, as routinely recommended. For each clinical or environmental sample, the screening of *S. capitis* NRCS-A was based on the specific color pattern of NRCS-A colonies after a 5-day period of incubation on MRSA Brilliance Agar (Oxoid®), and species identification was confirmed by MALDI-TOF MS, as previously described [9]. This chromogenic-based method has demonstrated an excellent sensitivity (100%) and a 94% specificity for the detection of NRCS-A that is why no additional genetic analysis were performed on the identified isolates.

### Vaginal colonization

A vaginal colonization with *S. capitis* NRCS-A was screened by testing anonymized vaginal samples from pregnant women consecutively received in our lab during a 3 weeks-period for the detection of Group B *Streptococcus* carriage.

### Carriage in caregivers

Nasal and hand samples were performed in voluntary caregivers working in the NICU. Each individual was sampled after 1 day working inside the setting and then after a wash-out period of at least 5 days from the NICU to distinguish a chronic carriage from a temporary colonization during working day.

### Environmental study

To determine the dissemination of *S. capitis* around patients, several standardized samples (*n* = 33 per patients, see details in Additional file 1: Table S1a) were collected in the close environment of hospitalized neonates. The environment of three “cases” i.e. neonates with *S. capitis* isolated in at least one blood culture in the previous 5 days and considered as truly infected by physician in charge of the patient, was sampled, and compared with the environment of three “controls”, defined by the absence of *S. capitis* in blood cultures since their birth.

To identify the possible environmental niches of the clone NRCS-A within this NICU, we screened weekly during six consecutive weeks the presence of *S. capitis* NRCS-A, in 23 sites inside the NICU, including care areas, relaxation and offices (see details in Additional file 1: Table S1b). Of note, the different surfaces inside the ward are routinely cleaned twice a day by the hospital cleaning staff, which is trained for such practices. The procedure of cleaning is performed in accordance with the recommendations of the hospital hygiene team.

### Procedures of disinfection of the incubators

The efficacy of the disinfection procedure on the eradication of *S. capitis* was tested by sampling incubators before and after disinfection (nine sites per incubator, detailed in Additional file 1: Table S1c). Incubators used in the unit are Giraffe™ (General Electrics Healthcare, Limonest, France) equipped with integrated scale, which allows for a daily body weight measure even in very sick and tiny infants. In this setting, incubators are changed every 10 days or earlier if the neonate did not need it any more. Between two patients, the incubators are disinfected using a 20-min disinfectant immersion bath (ANIOSURF Premium, Anios® including didecyldimethylammonium chlorure 82 mg/g, chlorhexidine digluconate 5 mg/g and polyhexamethylene biguanide chlorhydrate 0.24 mg/g; final dilution 0.25%), as recommended by the manufacturer. Some parts of the incubators, especially the scale and the mattress, that cannot be immerged, are handly disinfected using wet wipes impregnated with the same disinfectant solution. Finally, to detect a putative source of early colonization after disinfection procedure, the disinfection room was also screened for the presence of *S. capitis* (*n* = 29 samples, detailed in Additional file 1: Table S1d).

## Results

### Vaginal colonization

No *S. capitis* was identified in vaginal samples collected from 106 pregnant women.

### Carriage in caregivers

Twenty-one caregivers participated to the study. None of the nasal swabs was positive for *S. capitis*. Three of the 21 caregivers hand samples were positive but none of these three caregivers remained colonized after 5 days of “wash-out” period.

### Persistence in the NICU and dissemination around patients

*S. capitis* was isolated in the environment of cases and controls, in particular in incubators (Table 1). More than half samples (56/99, 57%) were positive around the three neonates with *S. capitis* sepsis. A third of samples (33/99, 33%) collected around the three uninfected infants were also positive. The environmental contamination was significantly higher around infected patients (*p* < 0.01, Chi2 test). During the six-week longitudinal study, a persistence of *S. capitis* NRCS-A within the NICU was observed, particularly on the equipment of the care area. About a third of samples (25/78, 32%) were positive, including computers, keyboards, chairs and phones. Offices and relaxation areas were rarely colonized by *S. capitis* (one positive sample out of 30).

**Table 1:** Detection of *S. capitis* NRCS-A among 33 specimens collected in the environment of *S. capitis* infected neonates (cases, *n* = 3) or non-infected neonates (controls, *n* = 3)

	Cases			Controls			
	A	B	C	A	B	C	
Incubator (*n* = 12)	9	11	10	1	10	9	
Equipment devoted to the patient (*n* = 11)	4	7	7	1	3	6	
Equipment of the setting (*n* = 10)	0	7	1	0	1	2	
Total	13	25	18	2	14	17	
Chi 2 comparison	56/99			33/99			*p* < 0,01

### Efficacy of the disinfection procedure of incubators

Because the incubators were the more frequently colonized surfaces around both cases and controls, we evaluated the efficacy of the disinfection procedures of incubators. A before versus after disinfection study was conducted for 16 consecutive incubators submitted to the routine disinfection procedure. All the incubators were colonized (i.e., at least one positive sample) with the clone NRCS-A before disinfection. Ten out of sixteen (63%) remained positive after the disinfection procedure. The mattress and the scale were the two most frequently positive samples (Table 2). Finally, the screening of the disinfection room did not reveal the presence of *S. capitis* NRCS-A except on the computer of this room.

**Table 2:** Detection of *S. capitis* NRCS-A on 9 locations of 16 incubators, before and after a disinfection procedure using disinfectant immersion bath

		N° of incubator															
	Sites of sampling	1	2	3	4	5	6	7	8	9	10	11	12	13	14	15	16
Before disinfection	Button “alarm off”	–	–	–	–	–	+	–	–	–	–	–	–	+	–	–	+
	Handle 1	+	–	–	–	–	–	–	–	–	–	–	+	–	–	–	–
	Handle 2	+	–	+	+	–	–	+	–	–	–	–	–	+	–	+	+
	Handle 3	–	–	–	+	+	–	+	–	+	–	–	–	–	–	–	+
	Handle 4	+	–	–	+	–	–	+	+	–	–	–	+	–	–	+	–
	Window	–	+	–	–	+	–	+	–	+	–	+	–	–	–	+	+
	Interstices around the scale	+	+	+	+	+	–	+	+	+	–	+	+	+	–	–	+
	Underside of the scale	+	–	+	–	+	+	–	–	+	+	–	+	+	+	–	–
	Mattress	+	–	–	+	+	+	+	+	+	+	–	+	+	+	–	+
	Total positive samples (n)	6	2	3	5	5	3	6	3	5	2	2	5	5	2	3	6
After disinfection	Button “alarm off”	–	–	–	–	–	–	–	–	–	–	–	–	–	–	–	–
	Handle 1	–	–	+	–	–	–	–	–	–	–	–	–	–	–	–	–
	Handle 2	–	–	+	–	–	–	–	–	–	–	–	–	–	–	–	–
	Handle 3	–	–	–	–	–	–	–	–	–	–	–	–	–	–	–	–
	Handle 4	+	–	+	–	–	–	–	–	–	–	–	–	–	–	–	–
	Window	–	–	+	–	–	–	–	–	–	–	–	–	–	–	–	–
	Interstices around the scale	+	–	–	+	–	–	–	–	+	–	–	+	–	–	–	–
	Underside of the scale	–	–	+	–	–	–	–	–	–	–	–	–	–	–	–	–
	Mattress	–	–	+	–	–	–	+	+	–	+	–	–	+	–	–	+
	Total positive samples (n)	2	0	6	1	0	0	1	1	1	1	0	1	1	0	0	1

## Discussion

The present study aimed to determine the potential sources and reservoirs within a NICU of the clone *S. capitis* NRCS-A, a major worldwide endemic pathogen involved in neonatal sepsis.

First, we tested the hypothesis of the vaginal flora as the source of maternal-fetal transmission of *S. capitis* NRCS-A. This hypothesis was proposed because the worldwide dissemination and the specific affinity for the neonates of the clone NRCS-A were reminiscent of the extensive diffusion of clonal Group B *Streptococcus* ST-17 causing neonatal sepsis [10]. However, our data did not support this mode of contamination. These results are not surprising because they are consistent with the data of previous studies showing that the onset of *S. capitis* sepsis was during the second week of life, which is not in favor of a maternal-fetal transmission [6, 7]. Furthermore, a large proportion of *S. capitis* colonized and infected neonates were born by caesarean-section, without contact between the vaginal flora and the baby [7]. Therefore, other potential sources of worldwide diffusion of *S. capitis* NRCS-A remain to be explored in the future.

In addition, we explored the potential reservoirs of *S. capitis* NRCS-A inside one NICU. In previous studies, incubators [11], ventilation system, balloons used for manual ventilation [12], diapers scale [13], stethoscopes and electronic devices [14] and mattresses [15] have been incriminated as environmental reservoirs of other pathogens, e.g. *Staphylococcus aureus, Bacillus cereus*, *Enterococcus faecalis, Klebsiella pneumoniae*. Concerning the species *S. capitis*, Gras-Le Guen et al. reported in 2002 an outbreak of *S. capitis* inside a NICU and identified almond oil bottles used for routine skin care as a possible reservoir [16]. However, in their study no other potential reservoirs had been tested. Recently, Carter et al. isolated *S. capitis* in several environmental sites inside one NICU, notably on stethoscopes and incubators [17]. In our present study, results showed that the circulation of the clone in the NICU predominated in the care area and on the incubators. Interestingly, none of the sampled sites was constantly positive for *S. capitis*, suggesting the absence of a single environmental niche inside the NICU. A high frequency of colonization of surfaces around infected but also non infected patients has been shown and could constitute the source of contamination for neonates. However, the colonization status of the patients whose the environment was sampled was not known so it is not possible to determine if the colonized environment leads to the colonization of the patient or if the colonization of the patient represents the source of transmission of *S. capitis* to his close environment. This last situation is conceivable since a previous study conducted in the same NICU has revealed a high frequency of stool colonization by *S. capitis* NRCS-A in hospitalized neonates [7]. Further studies are needed to better understand the cycle of contamination of the neonates inside the NICU. Especially a better approach could be to prospectively determine if an environmental contamination lead to previously un-colonized neonates being colonized.

The frequent colonization of the incubators can be explained by the lack of effectiveness of the disinfection protocol since in our study 62.5% of incubators remained colonized by *S. capitis* after the disinfection procedure. Because we cannot exclude an early recolonization of the incubators after disinfection which could be a bias in our before/after study, we screened the presence of *S. capitis* inside the disinfection room. Only one sample (computer keyboard) was positive for *S. capitis*. The protocol established in this ward is to systematically hand rubbing with an hydro alcoholic solution (available at this point of care) before touching incubators. It is unlikely that the disinfection staff could touch computer then incubators without this precaution, however hand hygiene compliance rate in the NICU has to be further evaluated.

The partial failure of the disinfection of incubators may be explained by a decreased susceptibility towards disinfectant molecules in *S. capitis* isolates, as reported in previous studies [17, 18]. Furthermore, in our study, the two most frequent sites of incubators which remained colonized after disinfection were the mattress and the interstices around the scale, which are two non-immersive parts of incubators. That is why, in addition to a possible resistance to disinfectant molecules, we suggest that technical difficulties in reaching the recesses of incubators and the seams of mattress could also have limited the antiseptic action and could have favored the persistence of bacteria, perhaps because of biofilm formation and/or seal colonization. This illustrates the gap between necessary evolution of care practices and medical devices (here, integration of scales into the incubator that is necessary for the care of very low birth weight infants) and the need to adapt maintenance of these devices. Hence, further work is needed to evaluate efficacy of other antiseptic molecules or other method of disinfection in order to optimize the disinfection procedure. In particular, a method based on steam could be of major interest. This method has been previously reported to have a good efficacy in some NICUs [19] and has been associated with the eradication of vancomycin-resistant *Enterococcus* in an Australian NICU [20]. More recently the implementation of steam disinfection of incubators has been associated with a significantly decreased incidence of *S. capitis* NRCS-A in one NICU [21].

Finally, we screened caregivers as a potential source of contamination due to *S. capitis* carriage, as it has already been reported for other staphylococci species [22]. Our results suggest that there is no chronic carriage of *S. capitis*. The hand colonization in some caregivers is likely related to a passive hand contamination due to iterative contacts with colonized surfaces and/or patients inside the NICU and it could participate to the inter-patient transmission, in case of defects in standard hygiene precautions notably hand washing procedures. This hypothesis has to be confirmed in future study.

A major limitation of our study is its single-center design, which limits the scope of our findings. Moreover, the environment of only three cases and three controls was sampled to screen *S. capitis*, due to technical and time constraints. A future study including several NICU settings could be of interest to confirm our results and increase the number of samples. Moreover such study will allow for a comparison of several NICU practices (disinfection procedures, antiseptics use, etc.).

## Conclusions

Taken together, our results lead to hypothesize a model of persistence and transmission of the *S. capitis* NRCS-A clone once introduced into a NICU. The ineffectiveness of disinfection procedures and possible decreased susceptibility to disinfectant molecules could contribute to the persistence of this clone inside the setting. Our findings are of major concern because they suggest that neonates could be housed in incubators still colonized by *S. capitis* NRCS-A, exposing them to a potential risk of subsequent colonization and infection. The optimization of the disinfection practices used in NICUs and in particular the investigation of steam efficacy, appear as key points to limit the spread of the clone inside a setting and to better manage outbreaks involving *S. capitis* in NICUs. Finally, for NICUs that are still free of *S. capitis* NRCS-A, adapted measures need to be implemented as soon as a first isolate is detected to immediately stop the implantation and dissemination of the clone into the setting.

## Supplementary information

**Additional file 1. **Locations for the screening of *S. capitis* NRCS-A: A. around the 3 infected patients and the 3 non-infected patients; B. collected weekly during 6 weeks inside NICU; C. on each incubator before and after disinfection procedure; D. inside the disinfection room.

## Data Availability

The datasets used and/or analysed during the current study are available from the corresponding author on reasonable request.

## Abbreviations

CoNS: Coagulase negative staphylococci
NICU: Neonatal intensive care units
