# Supplementary material for: Sources and reservoirs of Staphylococcus capitis NRCS-A inside a NICU
Source: Antimicrob Resist Infect Control. 2019 Oct 17;8:157. doi: 10.1186/s13756-019-0616-1 (PMC6798403; doi:10.1186/s13756-019-0616-1)
Supplement: Supplementary file 1 — Additional file 1. Locations for the screening of S. capitis NRCS-A: A. around the 3 infected patients and the 3 non-infected patients; B. collected weekly during 6 weeks inside NICU; C. on each incubator before and after disinfection procedure; D. inside the disinfection room. [file 13756_2019_616_MOESM1_ESM.docx]

**Additional file 1: Locations for the screening of *S. capitis* NRCS-A: A. around the 3 infected patients and the 3 non-infected patients; B. collected weekly during 6 weeks inside NICU; C. on each incubator before and after disinfection procedure; D. inside the disinfection room.**

A.

| **Locations of sampling** | | **Number of samples** |
| --- | --- | --- |
| **Incubator** |  |  |
|  | windows | 2 |
|  | handles | 2 |
|  | mattress | 4 |
|  | button "alarm off" | 2 |
|  | blanket above the incubator | 2 |
| **Equipment devoted to the patient** | |  |
|  | nursing trolley | 4 |
|  | respirator | 3 |
|  | monitoring device | 3 |
|  | hydroalcoolic solution piston | 1 |
| **Equipment of the setting** | |  |
|  | ultrasonic probe | 2 |
|  | ultrasond machine keyboard | 2 |
|  | computer mouse | 2 |
|  | computer keyboard | 2 |
|  | mouse pad | 2 |
| **TOTAL** |  | **33** |

B.

| **Care area** |  | **Equipment of the setting** | |
| --- | --- | --- | --- |
| Nursing area |  |  | ultrasonic probe |
|  | computer mouse |  | ultrasond machine keyboard |
|  | computer keyboard |  | handle of the Xray machine |
|  | chair |  | button of the Xray machine |
|  | phone |  | electrocardiograph |
|  | interphone |  |  |
|  | bench of drug preparation | **Relaxation area** |  |
|  | diaper scale |  | door of the relaxation room |
|  | light switch |  | table |
| Medical office inside the setting | |  | coffee machine |
|  | computer mouse |  | fridge |
|  | computer keyboard |  | door of the changing room |
|  | chair |  |  |
|  | phone |  |  |
|  | mobile phone of the resident |  |  |

C.

| **Sites of sampling** | **Number of samples** |
| --- | --- |
| windows | 1 |
| handles | 4 |
| mattress | 1 |
| button "alarm off" | 1 |
| scale | 2 |
| **TOTAL/incubator** | **9** |

D.

| **Sites of sampling** |  | **Number of samples** |
| --- | --- | --- |
| **Computer area** |  |  |
|  | computer keyboard and mouse | 1 |
|  | phone | 1 |
|  | light switch | 1 |
|  | whiteboard | 1 |
|  | crank handle of the shutter | 4 |
| **Disinfection area** |  |  |
|  | golves | 3 |
|  | sink | 2 |
|  | water mixer | 2 |
|  | tap | 2 |
|  | cupboard handle | 1 |
|  | cupboard door | 2 |
|  | cupboard inside | 2 |
|  | tiles | 2 |
|  | tile joint | 2 |
| **Incubator during disinfection procedure** | |  |
|  | scale | 1 |
|  | mattress | 1 |
|  | handle | 1 |
| **TOTAL** |  | **29** |
